# Cellular apoptosis susceptibility (CSE1L/CAS) protein in cancer metastasis and chemotherapeutic drug-induced apoptosis

**DOI:** 10.1186/1756-9966-29-110

**Published:** 2010-08-11

**Authors:** Cheng-Jeng Tai, Chung-Huei Hsu, Shing-Chuan Shen, Woan-Ruoh Lee, Ming-Chung Jiang

**Affiliations:** 1 Section of Hematology-Oncology, Department of Medicine, Taipei Medical University and Hospital, Taipei, Taiwan; 2 Department of Nuclear Medicine, Taipei Medical University and Hospital, Taipei, Taiwan; 3 Graduate Institute of Medical Science, Taipei Medical University, Taipei, Taiwan; 4 Department of Dermatology, Taipei Medical University and Hospital, Taipei, Taiwan

## Abstract

The cellular apoptosis susceptibility (CSE1L/CAS) protein is highly expressed in cancer, and its expression is positively correlated with high cancer stage, high cancer grade, and worse outcomes of patients. CSE1L (or CAS) regulates chemotherapeutic drug-induced cancer cell apoptosis and may play important roles in mediating the cytotoxicities of chemotherapeutic drugs against cancer cells in cancer chemotherapy. CSE1L was originally regarded as a proliferation-associated protein and was thought to regulate the proliferation of cancer cells in cancer progression. However, the results of experimental studies showed that enhanced CSE1L expression is unable to increase proliferation of cancer cells and CSE1L regulates invasion and metastasis but not proliferation of cancer cells. Recent studies revealed that CSE1L is a secretory protein, and there is a higher prevalence of secretory CSE1L in the sera of patients with metastatic cancer. Therefore, CSE1L may be a useful serological marker for screening, diagnosis and prognosis, assessment of therapeutic responses, and monitoring for recurrence of cancer. In this paper, we review the expression of CSE1L in cancer and discuss why CSE1L regulates the invasion and metastasis rather than the proliferation of cancer.

## Background

Cancer is a disease in which a group of cells in the body displays uncontrolled proliferation, invasion, and sometimes metastasis. Malignant cancers are known by their ability to escape from their original location and metastasize to the lymph nodes or other organs. Metastases are the main cause of cancer mortality; therefore diagnoses of metastatic cancer are critical for making therapeutic decisions. Non-metastatic tumors are usually treatable by surgical resection. For patients with cancer that has spread or metastasized, radiation, chemotherapy, or a combination of chemotherapy and radiation can be offered as treatment. Diagnosing cancer metastasis by assaying the level of serological markers of patients is relatively non-invasive. Serum markers that can detect cancer metastasis should be highly useful for screening, diagnosis, prognosis, assessment of therapeutic responses, and monitoring for recurrence of cancer and thus can provide information for taking medical practice to new levels of precision [1,2].

CSE1L/CAS, the cellular apoptosis susceptibility protein, was identified in a studying of an antisense cDNA fragment that is capable of causing MCF-7 human breast cancer cells resistant to apoptosis induced by bacterial toxins such as *Pseudomonas *exotoxin, diphtheria toxin, and tumor necrosis factor [3]. *CSE1L *is the human homologue of the yeast chromosome segregation gene, *CSE1*, and it encodes a 971-amino acid protein with an approximately 100-kDa molecular masses distributing in the cytoplasm and nuclei of cells [4]. CSE1L can associate with microtubules and mitotic spindles, which are cellular organelles for cell mitosis; thus, CSE1L was speculated to play a role in cancer cell proliferation, and was regarded as a proliferation-associated protein in 1996 [5,6]. Since then many pathological reports demonstrated that the expression of CSE1L in cancer is related to cancer proliferation [6-10], although there is no experimental studies to show that increased CSE1L expression in cancer cells can enhance the proliferation of cancer cells. CSE1L is highly expressed in cancer; thus, if CSE1L plays a role in cancer cell proliferation during cancer development, increased CSE1L expression in cancer cells should be able to increase the proliferation of cancer cells. Our recent study showed that increased CSE1L expression in MCF-7 human breast cancer cells was unable to stimulate cell proliferation [11]. Increased CSE1L expression was also unable to increase the proliferation of other cancer cells including HT-29 human colorectal cancer cells, Hep G2 human hepatocarcinoma cells, 293 kidney cancer cells, and B16-F10 mouse melanoma cells (unpublished data). The results of our study further showed that CSE1L enhanced the invasion and metastasis of B16-F10 cancer cells in animal metastasis studies [11].

CSE1L is a cellular apoptosis susceptibility protein and it is highly expressed in various cancers; our recent studies showed that CSE1L plays an important role in regulating cancer cell apoptosis induced by chemotherapeutic drugs [12,13]. Therefore, CSE1L may be a target for developing strategies to improve the efficacy of cancer chemotherapy as well as for screening more potent anticancer reagents.

### CSE1L in chemotherapeutic drug-induced cancer cell apoptosis

Apoptosis (or programmed cell death) plays an important role in mediating apoptotic stimuli including chemotherapeutic drug-induced cell cytotoxicity [14]. CSE1L is a cellular apoptosis susceptibility protein, and CSE1L-mediated cancer cell apoptosis was first investigated by Brinkmann *et al*. using a vector expressing antisense *CSE1L *cDNA. Their results showed that CSE1L mediated apoptosis induced by *Pseudomonas *exotoxin, diphtheria toxin, and tumor necrosis factor but did not mediate apoptosis induced by ricin, cycloheximide, staurosporine, or etoposide, a cancer chemotherapeutic drug. Therefore, CSE1L-mediated apoptosis was thought to be limited to selected apoptotic stimuli such as adenosine diphosphate (ADP)-ribosylating toxins and tumor necrosis factor [3,15]. CSE1L is essential for cell survival, and severe depletion of CSE1L can cause cell death [16]. Those studies used antisense *CSE1L *cDNA to reduce the cellular CSE1L level; hence the results of their studies might have been a result of those transfected cells expressing not very low levels of CSE1L. Also, they only tested the cancer chemotherapeutic drug, etoposide. An apoptosis-regulating protein should not only regulate apoptosis induced by just ADP-ribosylating toxins and tumor necrosis factor. CSE1L is highly expressed in cancer; therefore enhancing CSE1L expression rather than reducing CSE1L expression in cells is a more appropriate way to study CSE1L-mediated cancer cell apoptosis. We established HT-29 human colorectal cells and MCF-7 breast cancer cells stably transfected with the pcDNA-CSE1L vector, a eukaryotic expression vector carrying the full-length human *CSE1L *cDNA to study the effect of increased CSE1L expression on cancer cell apoptosis induced by chemotherapeutic drugs [12,13]. The chemotherapeutic drugs we tested including paclitaxel, doxorubicin, 5-fluorouracil, cisplatin, etoposide, and 4-OH-tamoxifen. Our results showed that CSE1L regulated cancer cell apoptosis induced by most of the chemotherapeutic drugs that we tested [12,13]. Increased CSE1L expression enhanced apoptosis induced by doxorubicin, 5-fluorouracil, cisplatin, and 4-OH-tamoxifen, but decreased apoptosis induced by paclitaxel in HT-29 cancer cells and MCF-7 cancer cells [12,13]. Therefore, CSE1L-mediated apoptosis is not limited to apoptosis induced by ADP-ribosylating toxins and tumor necrosis factor. Microtubules are the target of paclitaxel-induced cancer cell apoptosis [12], thus the expression of microtubule-associated protein may have an impact on cancer cell apoptosis induced by paclitaxel. For example, the expression of the microtubule-associated protein, caveolin-1, was reported to enhance paclitaxel-mediated apoptosis of MCF-7 cells [17]. Low expression level of the microtubule-binding protein, tau, was reported to enhance the sensitivity of human breast cancer to paclitaxel treatment [18]. CSE1L is also a microtubule-associated protein [5]. Paclitaxel treatment can block or prolong cells in the G2/M phase of the cell cycle during apoptosis induction [19], and to induce microtubule aster formation in apoptotic cells [20]. Cell cycle analyses showed that increased CSE1L expression inhibited paclitaxel-induced G2/M phase cell cycle arrest, and immunofluorescence studies showed that increased CSE1L expression inhibited paclitaxel-induced microtubule aster formation in cells [12]. Therefore, CSE1L might inhibit paclitaxel-induced apoptosis by affecting G2/M phase cell cycle arrest and microtubule aster formation induced by paclitaxel.

CPP32 (caspase-3) is one of the central apoptosis executioner molecules, and elevation of cleaved CPP32 is a sign of increased apoptosis [21]. Pathological studies showed that the expression of CPP32 was positively correlated with CSE1L expression in endometrial carcinoma (*p *= 0.008) [22]. Increased CSE1L expression can enhance both interferon-γ-induced CPP32 expression and the level of the cleaved CPP32 product, thereby inducing apoptosis of HT-29 cancer cells [23]. Therefore, the CPP32 apoptotic pathway is involved in CSE1L-mediated cancer cell apoptosis.

p53 is crucial in mediating cell apoptosis induced by various apoptosis-inducing stimuli, and most chemotherapeutic drugs exert their antitumor activity through a p53-dependent mechanism [24-28]. The activity of p53 is regulated by both the protein abundance and post-translational modifications of preexisting p53 molecules [29,30]. CSE1L was recently shown to associate with a subset of p53 target promoters, and reduced CSE1L expression decreased 53-mediated transcription and thus lowered apoptosis [31]. Our studies showed that increased CSE1L expression can enhance doxorubicin-induced p53 accumulation [12,13]; therefore, CSE1L regulates p53 protein accumulation induced by chemotherapeutic drugs. Other studies of ours also showed that interferon-γ treatment increased CSE1L expression in cancer cells [23] and interferon-γ co-treatment enhanced doxorubicin-induced p53 accumulation of Hep G2 hepatoma cells [32]. Thus, interferon-γ may increase doxorubicin-induced p53 accumulation by modulating CSE1L expression. CSE1L is highly expressed in cancer, and the results of our studies suggest that CSE1L plays a role in regulating p53 accumulation induced by chemotherapeutic drugs. Therefore, CSE1L may play an important role in mediating the cytotoxicities of chemotherapeutic drugs against cancer cells in cancer chemotherapy. Also, CSE1L may be a target for developing strategies to improve the efficacy and outcomes of cancer chemotherapy.

### CSE1L expression in cancer

CSE1L is highly expressed in various cancer types, and its expression level is positively correlated with high tumor stage, high tumor grade, and worse outcomes of cancer patients. The *CSE1L *gene is located on chromosome 20q13, a region frequently harbors amplifications that correlate with cancer aggression [33-35]. The copy number of the *CSE1L *gene is increased in breast, colon, and bladder cancer cell lines [36]. An array-based comparative genomic hybridization study showed high-frequency amplifications of the *CSE1L *gene in nasopharyngeal carcinomas [37] and in medulloblastomas [38]. The results of array-based comparative genomic hybridization showed that 57.1% of the glioblastoma multiforme cases had high-frequency amplification of the *CSE1L *gene [39]. Idbaih *et al*. investigated a series of 16 low-grade gliomas and their subsequent progression to higher-grade malignancies using a one-megabase bacterial artificial chromosome (BAC)-based array comparative genomic hybridization technique, and reported that the *CSE1L *gene was associated with the progression of gliomas [40]. The results of another study using microarray-based detection showed that CSE1L was highly expressed in nasopharyngeal carcinomas [41]. Combined cytogenetic, array-based comparative genomic hybridization studies and expression analyses also showed that CSE1L was significantly overexpressed in advanced prostate cancer xenografts [42].

The results of a pathological study showed that expression of CSE1L was not detected in normal hepatocytes, while strong CSE1L expression was detected in hepatocellular carcinoma [10]. Another study showed that the immunohistochemical staining intensity score of CSE1L was significantly higher in human hepatocellular carcinoma than in non-tumor tissue (*p *< 0.05) [43]. In breast cancer, benign lesions of the breast showed weak CSE1L staining, while 70% - 90% of breast tumor cells were heavily stained for CSE1L [9]. In serous ovarian carcinoma, moderate to strong immunostaining of CSE1L was observed in 34 of 41 cases (83%) of serous carcinomas, and CSE1L immunoreactivity was positively related to the frequency of apoptotic bodies (*p *= 0.0170), the tumor grade (*p *= 0.0107), and adverse outcomes (*p *= 0.0035) [44]. Peiro *et al*. reported that CSE1L protein reactivity was present in 100% of 69 ovarian carcinomas, and a significant reciprocal correlation was observed between high levels of CSE1L and the histological type, FIGO (International Federation of Obstetrics and Gynecology) stage III and grade 3, residual tumors of > 2 cm, and 20q13.2 (ZNF217 gene) amplification (> four copies in > 20% cells) [45]. A tissue array study composed of 244 serous ovarian tumors of different grades (0-3) and stages (I-IV) showed a higher expression of CSE1L in poorly compared to highly differentiated invasive ovarian tumors [46].

An analysis of 89 endometrial carcinomas and 56 samples of non-neoplastic adjacent endometrium showed that CSE1L was expressed in 93% of endometrial carcinomas neoplastic tissues, while lower levels of CSE1L expression were observed in the adjacent endometrium compared to the carcinomas (*p *= 0.003). Also, CSE1L expression was higher in grade 3 tumors (*p *= 0.002) [22].

Boni *et al*. studied the expression of CSE1L in 27 control benign and 55 malignant melanocytic lesions (including 32 primary and 23 metastatic lesions), and their results showed that only 13 of the 27 benign melanocytic lesions stained positive for CSE1L [7]. However, 5 of 7 lentigo maligna melanomas, 11 of 12 superficial spreading melanomas, and all acrolentiginous (*n *= 7) and nodular (*n *= 6) melanomas showed medium to high intensity immunoreactivity for CSE1L staining [7]. All metastatic melanomas (*n *= 23) they studied showed strong CSE1L staining [7]. Also, CSE1L detection in clinical stages according to the International Union Against Cancer (UICC) showed an increase from 43% ± 34% CSEL-positive cells in stage I, to 53% ± 26% in stage II, 68% ± 24% in stage III, and 72% ± 24% in stage IV [7].

In normal lymphoid tissue and malignant lymphomas, low-grade non-Hodgkin's lymphoma revealed weak CSE1L staining, with 10% to 60% of all cells positive [6]. In contrast, highly malignant non-Hodgkin's lymphoma and malignant cells of Hodgkin's disease displayed very strong CSE1L positivity, with staining of up to 80% of atypical cells [6]. CSE1L was recently shown to be expressed in brain pilocytic astrocytomas [47]. The expression of CSE1L was also reported to be higher in the primary and metastatic human colorectal carcinoma compared to the normal colon mucosa (*p *< 0.0001) [48]. Recent study also showed that the distribution CSE1L in the epithelial glands of neoplastic colorectal epithelium was related to the malignance of colorectal cancer [49].

The pathological studies showed amplification of the *CSE1L *gene or high expression of CSE1L protein in various cancer types including hepatocellular carcinomas, endometrial carcinomas, cutaneous melanomas, lymphomas, ovarian carcinomas, breast carcinomas, prostate cancers, nasopharyngeal carcinomas, medulloblastomas, glioblastomas, and colorectal carcinomas. The pathological studies also showed that the expression of CSE1L was positively correlated with a higher cancer stage and higher cancer grade, indicating that CSE1L plays an important role in cancer development and progression.

### CSE1L is unable to increase cancer cell proliferation

Cancer cells are characterized by their uncontrolled proliferative abilities. CSE1L is the human homologue of the yeast chromosome segregation gene, *CSE1 *[4]. Mutation of the yeast *CSE1 *was shown to lead to defects in both chromosome segregation and B-type cyclin degradation; therefore a role of yeast *CSE1 *in facilitating the mitotic phase (not the S phase) of yeast replication was described [50,51]. Another study by Yu *et al*. reported that depletion of *CSE1 *resulted in a defect in the S-phase progression of yeast; therefore they demonstrated that *CSE1 *plays a role in DNA replication during yeast proliferation [52]. It should be noted, however, that their studies were based on *CSE1 *mutation or depletion and did not include an experiment to see the effect of increased CSE1 expression on yeast replication. Moreover, an immunofluorescence study of the distribution of human CSE1L in cells showed that CSE1L was associated with microtubules and mitotic spindle of mitotic cells; hence CSE1L was first suggested by Scherf *et al*. to play a role in promoting the mitotic phase of the cell cycle, and thus CSE1L was assumed to be able to increase the proliferation of human cells [5]. Another study by Ogryzko *et al*. reported that transient transfection of vectors carrying the antisense *CSE1L *cDNA into HeLa human cervical cancer cells interfered with cell mitosis [53]. Because CSE1L is highly expressed in various cancers, CSE1L was thus regarded as a proliferation-associated protein and was thought to play a role in tumor proliferation during cancer development and progression [8,54]. Consequently, many pathological studies reported that the expression of CSE1L was positively correlated with tumor proliferation, and the role of CSE1L in cancer progression was to increase tumor proliferation [6-10], although there are no experimental studies showing that increased CSE1L expression in cancer cells can increase cancer cell proliferation.

We amplified the full-length *CSE1L *cDNA from human cells and cloned it into the pcDNA3.1 eukaryotic-expressing vector to obtain the pcDNA-CSE1L vector to study the effect of increased CSE1L expression on cancer cell proliferation [11,55]. Our results showed that increased CSE1L expression in HT-29 cells did not increase cell proliferation, but on the contrary, increased CSE1L expression decreased the proliferation of HT-29 cells [55]. The HT-29 human colorectal cancer cell line is a special cell line as it easily becomes polarized in culture [56]. The formation of cell polarity is related to cell proliferation, and loss of apical-basal cell polarity can increase cell proliferation [57]. Increased CSE1L expression in HT-29 cells stimulated polarization of HT-29 cells [58]. Hence, we thought that the decrease in cell proliferation of pcDNA-CSE1L vector-transfected HT-29 cells might be a result of polarization of HT-29 cells induced by increased CSE1L expression, and not a result of increased CSE1L expression that directly decreased the proliferation of HT-29 cells [55]. Nevertheless, our other studies showed that although increased CSE1L expression was unable to induce polarization of MCF-7 cancer cells as it did in HT-29 cells, enhanced CSE1L expression in MCF-7 cells still decreased but not increased the proliferation of MCF-7 cells [11]. Therefore, CSE1L is unable to stimulate cancer cell proliferation.

CSE1L may be necessary for the M phase cell cycle progression of cells, thus a reduction in the CSE1L level can lead to a defect in chromosome segregation in the mitotic cell-cycle phase. However, it is quite impossible that high expression of CSE1L in cancer cells can enhance chromosome segregation at the mitotic phase of cells and thus increase cancer cell proliferation. First, the key step that determines the rate limitation for cell proliferation is mainly at the G1-S phase of the cell cycle rather than at the M phase [59]. Second, CSE1L is associated with mitotic spindles and functions in the mitotic spindle checkpoint; therefore high expression of CSE1L in cancer cells may halt the progression of mitosis until the cells are truly ready to divide. The p53 protein also plays a role in activating cell-cycle checkpoints, and activation of p53 can stop cell-cycle progression at the cell-cycle checkpoints [60]. The involvement of CSE1L in the proliferation of cancer cells was also supported by a pathological study which reported that the expression of the Ki67 proliferation marker was significantly positively correlated with CSE1L in a study of malignant lymphomas; nevertheless, that study also showed that a significant fraction of CSE1L-positive malignant lymphocytes were Ki-67 negative [6]. Various oncogenes may be activated and various anti-oncogenes may be inactivated in tumors; the activated oncogenes and inactivated anti-oncogenes can stimulate the proliferation of cancer cells that highly express CSE1L. Therefore, a positive correlation between CSE1L and Ki67 expression in tumors is insufficient to conclude that CSE1L can stimulate cancer cell proliferation. CSE1L is an apoptosis susceptibility protein; hence increased CSE1L expression can cause cells to be susceptible to apoptosis, let alone to stimulate cell proliferation. In our studies, MCF-7 cells and HT-29 cells transfected with CSE1L-expressing vectors were prone to apoptosis, and exhibited a relatively lower cell growth rate as compared to those of the control vector-transfected cells [11]. Recently, CSE1L was shown to be associated with a subset of p53 target promoters, and reduced CSE1L expression decreased 53-mediated transcription and lowered apoptosis [31]. p53 is known to be able to promote the expression of cell-cycle arrest target genes while enhancing the transactivation of proapoptotic genes [61]. Therefore, that report further suggested that although CSE1L definitely plays an important role in cancer progression, it does not stimulate cancer proliferation. Finally, CSE1L is highly, not barely, expressed in cancer. However, studies reporting that human CSE1L (also yeast CSE1) is associated with cell proliferation were only based on the effect of CSE1L reduction or CSE1 deletion on the growth of human or yeast cells. Therefore, it is inappropriate to use the results of CSE1L reduction experiments to assume that CSE1L can stimulate or increase cancer cell proliferation and draw a conclusion that the role of CSE1L in cancer development is to stimulate cancer proliferation.

### CSE1L enhances matrix metalloproteinase-2 secretion and increases cancer cell invasion

Increased CSE1L expression is unable to enhance the proliferation of cancer cells, thus CSE1L may promote cancer progression by other mechanisms. A pathological study by Brustmann *et al*. reported that the immunoreactivity of CSE1L was positively related to high cancer grade (*p *= 0.0107) and adverse outcomes (*p *= 0.0035) in serous ovarian carcinoma [44]. By studying 89 samples of endometrial carcinomas and 56 samples of the non-neoplastic adjacent endometrium, Peiro *et al*. reported that CSE1L expression was higher in grade 3 tumors (*p *= 0.002), and a shorter survival was observed for patients whose tumors contained > 50% of CSE1L-positive cells (*p *= 0.04) [22]. A tissue array study composed of 244 serous tumors of different grades (0-3) and stages (I-IV) showed a higher expression of CSE1L in poorly compared to highly differentiated invasive ovarian tumors [46]. The expression of CSE1L was correlated with advanced stages of melanomas and clinical stages according to the UICC which showed an increase from 43% ± 34% of CSE1L in stage I, to 53% ± 26% in stage II, 68% ± 24% in stage III, and 72% ± 24% in stage IV [7]. Heavy CSE1L staining was observed in all of the metastatic melanoma (*n *= 23) they studied [7]. The results of these pathological studies indicated that the expression of CSE1L was positively related to high cancer stage and worse outcomes of cancer patients. Metastasis is the main characteristic of high cancer stages and is also the main cause of cancer-related mortality. Therefore, CSE1L may regulate the invasion and metastasis of cancer.

CSE1L can associate with microtubules [4] and the nuclear-transport receptor, importin-α [62]. Hence, CSE1L was predicted to show granule-like staining in the perinuclear areas of cells due to its association with importin-α, or show microtubule-like staining due to its association with microtubules in immunofluorescence study. However, in a study of the distribution of CSE1L in cancer cells, we observed that in addition to granule-like staining in cytoplasm surrounding the perinuclear areas, CSE1L also showed vesicle-like staining in the protrusions of MCF-7 cells in immunofluorescence [63]. Cytoplasmic vesicles play important roles in regulating the exocytosis and secretion of cells [64]. The vesicle-like staining of CSE1L in cell protrusions indicates that CSE1L may play a role in regulating cell secretion. The protrusions of cancer cells also play a role in facilitating cancer cell invasion [65]. Furthermore, increased CSE1L expression was shown to increase the secretion of HT-29 cells [66]. These results suggest that CSE1L may regulate the secretion and invasion of cancer cells.

Extracellular matrix (ECM) surrounding tumor and ECM-degrading proteases secreted by tumor cells play crucial roles in modulating cancer metastasis [67-69]. Matrix metalloproteinases (MMPs), including MMP-2, are enzymes involved in the degradation of ECM, which show increased expression during cancer metastasis [70-76]. MMP-2 production can be regulated at the level of secretion [77]. Metastatic tumor cells often develop enhanced secretory abilities in order to enhance MMPs secretion, thereby enhancing their metastatic potential [78]. Double-staining immunofluorescence showed that CSE1L regulates the translocation and secretion of MMP-2-containing vesicles [11]. Matrigel-based invasion assays showed that enhanced CSE1L expression increased cell invasion, and reduced CSE1L expression inhibited the invasion of MCF-7 cancer cells [11]. Finally, animal tumor metastasis experiments showed that reduced CSE1L expression decreased the pulmonary metastasis of B16-F10 cells, a highly metastatic cancer cell line, in C57BL/6 mice [11,79]. Therefore, CSE1L regulates MMP-2 secretion and enhances the invasion of cancer cells.

### CSE1L is a secretory protein and there is a higher prevalence of secretory CSE1L in sera of patients with metastatic cancer

**Figure 1:**
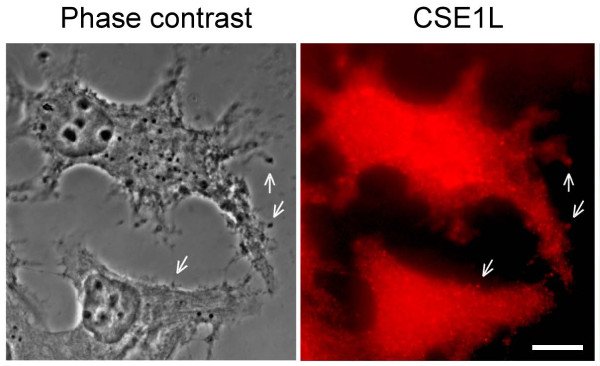
**CSE1L staining in vesicles surrounding the outside of cell membrane**. The distribution of CSE1L in MCF-7 cells was analyzed by immunohistochemistry with anti-CSE1L antibody. Note the vesicle-like staining of CSE1L in cell protrusions and positive staining of CSE1L in vesicles surrounding the outside of the cell membrane. The scale bar = 30 μm. The photo is derived from a figure in reference 63 [63].

CSE1L is highly expressed in cancer, and its expression level is well correlated with advanced cancer stage and worse patient outcomes. Therefore, CSE1L may play an important role in cancer progression. CSE1L is a microtubule-associated protein [4]. Our recent study showed that the association of CSE1L with microtubules is related with protrusion extension and migration of MCF-7 breast cancer cells [80]. In the immunofluorescence study, CSE1L was colocalized with MMP-2 in vesicles surrounding the outside of the MCF-7 cell membranes [Fig 1; also see [63]]. Since MMP-2 is a secretory protein, these results suggest that CSE1L may be secreted together with MMP-2. In immunohistochemistry, positive CSE1L staining was observed in the gland lumen of different cancers including breast cancer and colorectal cancer [63]. The tumor microenvironment, or stroma, consists of ECM and plays an important role in regulating cancer metastasis [81,82]. Glands, the major epithelial components of tubular organs, mediate the passage and control of homeostasis by modifying secretion. Glands in cancer tissues also provide the metastatic cancer cells with a route for invasion to adjacent tissues or other organs [83]. Moreover, substances that are secreted from a gland lumen can ultimately reach blood vessels [84]. CSE1L staining in the gland lumen of metastatic cancer tissues indicate that CSE1L may be secreted by cancer tissues and CSE1L may be a secretory protein.

CSE1L as a secretory protein was assessed by immunoblotting with conditioned medium harvested from B16-F10 cancer cells, and the results showed that CSE1L was present in conditioned medium of serum-starved B16-F10 cells [63]. That result confirmed that CSE1L is a secretory protein. Serum samples collected from patients with metastatic cancer were assayed for the presence of secretory CSE1L in sera of patients with metastatic cancer. The results of immunoblotting also showed that secretory CSE1L is present in sera of patients with metastatic cancer [63]. The results of enzyme-linked immunosorbent assay (ELISA) showed that serum CSE1L was detected in 58.2% (32/55), 32.0% (8/25), and 12.1% (8/66) of patients with metastatic, invasive, and primary cancers, respectively [63]. Serum CSE1L was more prevalent in patients with metastatic cancer. The presence of secretory CSE1L in the sera of patients with metastatic cancer was not restricted to a specific cancer type. Analyses of serum samples from patients with metastatic cancer showed that serum CSE1L was detected in various cancer types including colorectal cancer, breast cancer, lung cancer, cervical cancer, bile duct cancer, esophageal cancer, ovarian cancer, oviduct omental cancer, and head and neck cancer [63,85]. Recent study also showed that CSE1L was present in cerebrospinal fluids of patients with intracerebral hemorrhage [86]. Therefore, CSE1L is a secretory protein, and there is a higher prevalence of secretory CSE1L in sera of patients with metastatic cancer.

## Conclusions

Metastasis is the main cause of cancer-related mortality; therefore the screening and diagnosis of metastatic cancer are important for cancer treatment [87-95]. CSE1L is highly expressed in various cancers especially high stage cancers, and thus it may play important roles in modulating the development and progression of cancer. CSE1L was previous regarded as a proliferation-associated protein and was thought to be associated with tumor proliferation in cancer progression. Experimental studies showed that increased CSE1L expression in cancer cells was unable to enhance cancer cell proliferation. CSE1L actually is a secretory protein associated with cancer metastasis, and CSE1L is more frequently detected in sera of patients with metastatic cancer than with primary cancer. Therefore, CAS may have clinical utility in metastatic cancer screening and diagnosis, and it may be a potential target for anti-metastasis therapy.

## Competing interests

The authors declare that they have no competing interests.

## Authors' contributions

CJT and MCJ wrote the paper. CHH, SCS, and WR L discussed and participated in paper writing. All authors read and approved the final manuscript.
